# A new species of *Polypedilum* (*Uresipedilum*) Oyewo & Sæther, 1998 from Zhejiang Province of Oriental China (Diptera, Chironomidae)

**DOI:** 10.3897/zookeys.320.5147

**Published:** 2013-07-31

**Authors:** Xiaolong Lin, Xin Qi, Ruilei Zhang, Xinhua Wang

**Affiliations:** 1 College of Life Science, Nankai University, Tianjin 300071, China; 2 College of Life Science, Taizhou University, Taizhou, Zhejiang 318000, China; 3 College of Fisheries and Life Science, Shanghai Ocean University, Shanghai, 201306, China

**Keywords:** Chironomidae, *Polypedilum* (*Uresipedilum*), key, new species, China

## Abstract

A new species of *Polypedilum (Uresipedilum)* Oyewo & Sæther, 1998, *Polypedilum (Uresipedilum) minimum* **sp. n.** is described as male. A key to adult males of the subgenus from China is presented.

## Introduction

*Polypedilum* is one of the largest chironomid genera containing about 440 described species. The larvae mostly occur in sediments, with a few species mining wood or grazing epilignic and epilithic surfaces (Cranston et al. 1989). At present, the genus *Polypedilum* comprises eight subgenera: *Polypedilum* Kieffer, 1912, *Pentapedilum* Kieffer, 1913, *Kribionympha* Kieffer, 1921, *Tripedilum* Kieffer, 1921, *Tripodura* Townes, 1945, *Uresipedilum* Oyewo & Sæther, 1998, *Cerobregma* Sæther & Sundal, 1999 and *Probolum* Andersen & Sæther 2010 (Sæther et al. 2010).

Sasa and Kikuchi (1995) proposed *Uresipedilum* for the *Polypedilum convictum* group sensu Niitsuma (1992), but they failed to designate the type species. Oyewo and Sæther (1998) validated the name by designating *Polypedilum (Uresipedilum) convictum* (Walker, 1856) as the type species. Zhang and Wang (2004) reviewed the subgenus on the basis of 14 species recorded in China. Sæther and Oyewo (2008) and Sæther et al. (2010) revised the subgenus around the world and transferred *Polypedilum (Uresipedilum) bullum* Zhang & Wang, 2004, *Polypedilum (Uresipedilum) pedatum excelsius* Townes, 1945 and *Polypedilum (Uresipedilum) simantokeleum* Sasa, Suzuki & Sakai, 1998 to the newly proposed subgenus *Probolum*. Up to date, *Uresipedilum* includes 46 known species.

The adult males of the subgenus *Uresipedilum* are separated from other subgenera by having the basal portion of the superior volsella much longer than wide, with an apicomedian projection without setae placed on the inner margin of the base and directed medially and without prominent inner projection; wing membrane without markings or setae and fore tibial scale nearly always without spur (Sæther et al. 2010).

Based on the material from Zhejiang Province of Oriental China, a new species is described and illustrated as male. A complemented key to adult males of *Polypedilum (Uresipedilum)* from China is presented.

## Materials and methods

The morphological nomenclature follows Sæther (1980) and the abbreviations of structures measured follow Qi et al. (2012). The material examined was slide-mounted, following the procedure by Sæther (1969). The specimen examined in this study is deposited in the College of Life Science, Nankai University, China.

## Taxonomy

### Polypedilum (Uresipedilum) minimum sp. n.

http://zoobank.org/3237A70B-1254-4EB7-969B-6FD4ACC1883D

http://species-id.net/wiki/Polypedilum_minimum

Figures 1–7

#### Diagnosis.

The male adult can be distinguished from known species of the subgenus by the following combination of characters: low AR (0.27); frontal tubercles present; fore tibial scale rounded; anal point broad; superior volsella strongly projected posteriorly, pointed at apex, without microtrichium; high HV (4.90).

#### Description.

Male adult (n = 1). Total length 1.47 mm. Wing length 0.89 mm. Wing length/length of profemur 2.98.

Coloration. Head, legs and abdomen yellow. Thorax yellow with brown vittae, postnotum and preepisternum.

**Figures 1–7.**
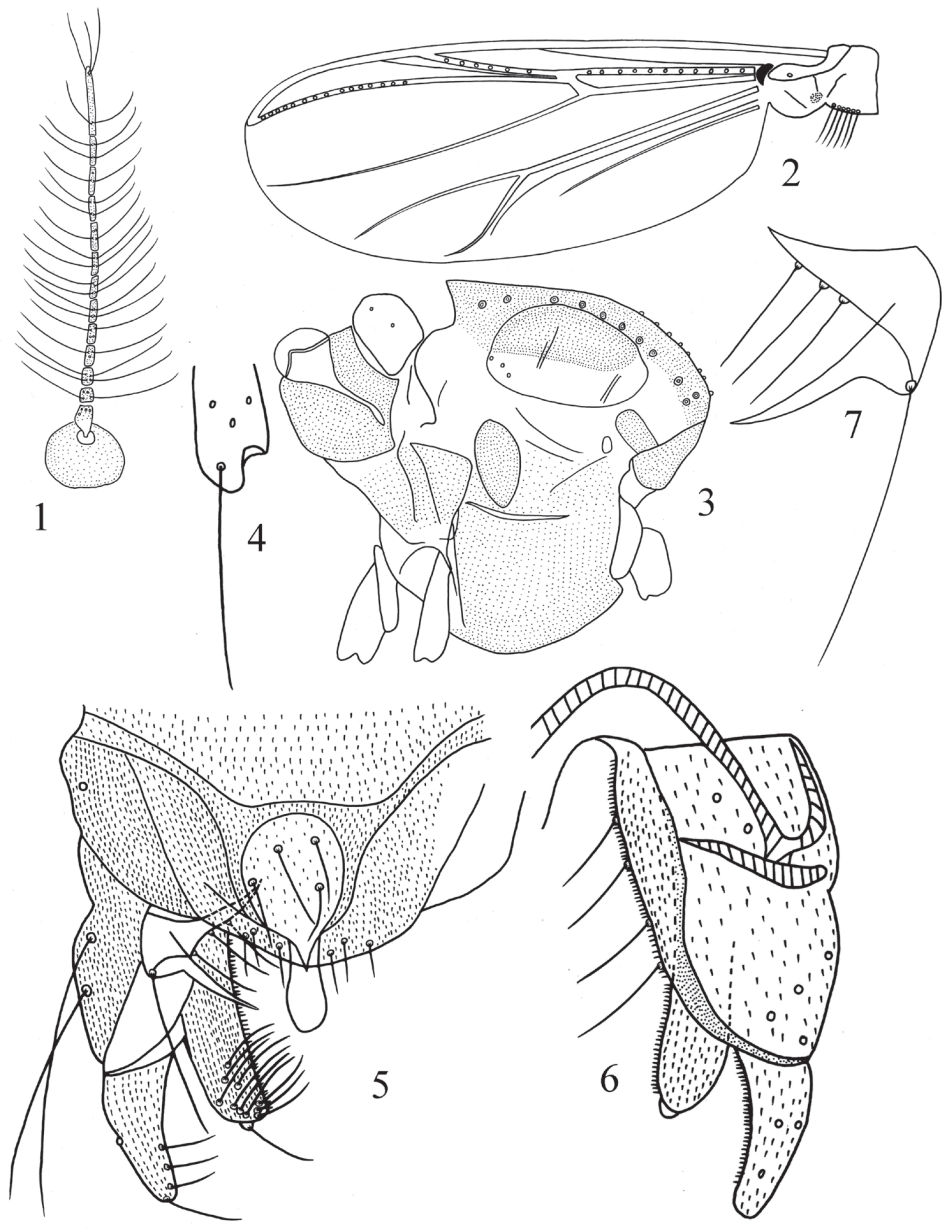
*Polypedilum (Uresipedilum) minimum* sp. n. **1** Antenna. **2** Wing. **3** Thorax **4** Fore tibia scale **5** Dorsal view of hypopygium **6** Ventral view of hypopygium **7** Superior volsella.

Head. AR 0.27. Antenna with 13 flagellomeres, ultimate flagellomere 93 μm long (Fig. 1). Frontal tubercles 38 μm long, 15 μm wide at base. Temporal setae 7, including 2 inner verticals, 4 outer verticals and 1 postorbital. Clypeus with 15 setae. Tentorium 70 μm long, 10 μm wide. Stipes 75 μm long, 10 μm wide. Palpomeres length (in μm): 18, 15, 38, 55, 103. L: 5^th^/3^rd^ 2.73.

Wing (Fig. 2). VR 1.54. Brachiolum with 1 seta, R with 11 setae, R_1_ with 6 setae, R_4+5_ with 15 setae. Squama with 6 setae.

Thorax (Fig. 3). Acrostichals 8; dorsocentrals 11; prealars 3. Scutellum with 4 setae.

Legs. Terminal scale (Fig. 4) of fore tibia rounded, 15 μm long, without spine. Spur of mid tibia 25 μm long, comb 10 μm long; unspurred comb 15 μm long. Spur of hind tibia 25 μm long, comb 10 μm long; unspurred comb 10 μm long. Apex of fore tibia 25 μm wide, of mid tibia 23 μm wide, of hind tibia 38 μm wide. Mid ta_1_ without sensilla chaetica. Lengths (in μm) and proportions of legs in Table 1.

**Table 1.** Lengths (in μm) and proportions of legs of *Polypedilum (Uresipedilum) minimum* sp. n.

	**p_1_**	**p_2_**	**p_3_**
fe	299	365	374
ti	215	251	308
ta_1_	317	165	194
ta_2_	143	75	120
ta_3_	115	60	115
ta_4_	85	38	65
ta_5_	40	38	50
LR	1.47	0.66	0.63
BV	2.17	3.70	2.50
SV	1.62	3.73	3.52
BR	3.60	4.33	5.14

Hypopygium (Figs 5–6). Tergite IX with 4 strong median setae. Laterosternite IX with 1 seta. Anal point broad, 18 μm long, with swollen, rounded apex. Phallapodeme 34 μm long; transverse sternapodeme 13 μm long. Gonocoxite 62 μm long, with 2 long setae. Superior volsella (Fig. 7) 16 μm long, with 3 basal inner setae and 1 strong apical seta, without microtrichium; apicomedial projection 17 μm long, pointed at apex. Inferior volsella 41 μm long, with 10 dorsal setae and 1 prominent apical seta. Gonostylus 30 μm long, apex blunt, with 3 setae along inner margin and 1 apical seta. HR 2.07. HV 4.90.

#### Type materials.

Holotype: adult male, China, Zhejiang Province: Jinhua City, Pan’an County, Dapanshan National Nature Reserve, 120.50°N, 29.00°E, 18.vii.2012, leg. Lin XL, sweep net.

#### Etymology.

From Latin *minimum*, little, referring to the small body length and antennal ratio.

#### Remarks.

The new species resembles *Polypedilum (Uresipedilum) obtusum* Townes, 1945, *Polypedilum (Uresipedilum) aviceps* Townes, 1945, *Polypedilum (Uresipedilum) infundibulum* Zhang & Wang, 2004, *Polypedilum (Uresipedilum) surugense* Niitsuma, 1992 and *Polypedilum (Uresipedilum) paraviceps* Niitsuma, 1992 in the general structure of hypopygium, but it can be separated by the superior volsella without microtrichium, which present in the latter species; the low antennal ratio (AR= 0.27), AR>1 in the latter species. The new species most resembles *Polypedilum (Uresipedilum) breviplumosum* Zhang & Wang, 2004 in the low antennal ratio (AR= 0.22–0.23), but it differs from the latter species by the following combination of characters in Table 2.

**Table 2.** Differences between *Polypedilum (Uresipedilum) minimum* sp. n. and *Polypedilum (Uresipedilum) breviplumosum* Zhang & Wang, 2004.

	***Polypedilum (Uresipedilum) minimum* sp. n.**	***Polypedilum (Uresipedilum) breviplumosum***
TL	1.47 mm	2.24–2.57 mm
WL	0.89 mm	1.43–1.59 mm
Setae on squama	6	9–12
Anal point	board	slender
Superior volsella	strongly projected posteriorly, bare, with 3 inner setae, pointed at apex	rounded at apex, covered with microtrichia
Fore tibial scale	rounded	pointed
HR	2.07	1.25–1.40
HV	4.90	2.57–2.73

Female and immature stages unknown.

### Key to adult males of *Polypedilum (Uresipedilum)* from China

**Table:** 

1	Anal point semicircular	2
–	Anal point triangular	5
2	Base of superior volsella without microtrichium	3
–	Base of superior volsella with microtrichia	4
3	AR 1.25–1.48, superior volsella without inner seta	*Polypedilum (Uresipedilum) infundibulum* Zhang & Wang, 2004
–	AR 0.27, superior volsella with 3 inner setae	*Polypedilum (Uresipedilum) minimum* sp. n.
4	Subapical tubercle of inferior volsella present	*Polypedilum (Uresipedilum) paraviceps* Niitsuma, 1992
–	Subapical tubercle of inferior volsella absent	*Polypedilum (Uresipedilum) surugense* Niitsuma, 1992
5	AR about 0.2, frontal tubercles present	*Polypedilum (Uresipedilum) breviplumosum* Zhang & Wang, 2004
–	AR>0.8, frontal tubercles absent	6
6	Anal point with several lateral setae	7
–	Anal point without lateral setae	8
7	Inner margin of superior volsella bulging	*Polypedilum (Uresipedilum) lateralum* Zhang & Wang, 2004
–	Inner margin of superior volsella medially constricted	*Polypedilum (Uresipedilum) dilatum* Zhang & Wang, 2004
8	Inferior volsella with large ventral apical process	*Polypedilum (Uresipedilum) prominens* Zhang & Wang, 2004
–	Inferior volsella without large ventral apical process	9
9	Fore tibial scale rounded	10
–	Fore tibial scale pointed	11
10	Base of superior volsella with 1–3 inner setae, projected posteriorly	*Polypedilum (Uresipedilum) convictum* (Walker, 1856)
–	Base of superior volsella with 4–5 inner setae, not projected posteriorly	*Polypedilum (Uresipedilum) crassiglobum* Zhang & Wang, 2004
11	Base of superior volsella without seta	*Polypedilum (Uresipedilum) medium* Zhang & Wang, 2004
–	Base of superior volsella with several setae	12
12	Superior volsella with 2–5 apical setae	*Polypedilum (Uresipedilum) cultellatum* Goetghebuer, 1931
–	Superior volsella with 1 apical seta	13
13	Apicomedial projection of superior volsella much shorter than base	*Polypedilum (Uresipedilum) basilarum* Zhang & Wang, 2004
–	Apicomedial projection of superior volsella much longer than base	*Polypedilum (Uresipedilum) xuei* Zhang & Wang, 2004

## Supplementary Material

XML Treatment for Polypedilum (Uresipedilum) minimum

## References

[B1] Cranston PS Dillon ME Pinder CV Reiss F (1989) The adult males of Chironominae (Diptera: Chironomidae) of the Holarctic region -Keys and diagnoses. In: Wiederholm T (Ed). Chironomidae of the Holarctic region. Keys and diagnoses: Part 3. Adult males. Entomologica Scandinavica Supplement 34: 353–532.

[B2] Kieffer JJ (1912) Tendipedidae (Chironomidae) (Dipt.). H. Sauter’s Formosa-Ausbeute. Supplementa entomologica 1: 27-43.

[B3] Kieffer JJ (1913) Nouveaux Chironomides (Tendipédides) d’Allemagne. Bulletin de la Société d’Histoire naturelle de Metz 28: 7-35.

[B4] Kieffer JJ (1921) Synopse de la tribu des Chironomariae (Diptères). Annales de la Société scientifique Bruxelles 40: 269-277.

[B5] Niitsuma H (1992) The *Polypedilum convictum* species group (Diptera, Chironomidae) from Japan, with descriptions of two new species. Japanese Journal of Entomology 60: 693-706.

[B6] Oyewo EA Sæther OA (1998) Revision of Afrotropical *Polypedilum* Kieffer subgenus *Uresipedilum* Sasa et Kikuchi, 1995 (Diptera: Chironomidae), with a review of the subgenus. Annales de Limnologie 34: 315–362. http://www.limnology-journal.org/download.php?file=%2FANL%2FANL34_03%2FS0003408898000284a.pdf&code=96e7063756d7391113924f0d065b8fde doi: 10.1051/limn/1998028

[B7] Qi X Lin XL Wang XH (2012) Review of *Dicrotendipes* Kieffer from China (Diptera: Chironomidae). Zookeys 183: 23-36. doi: 10.3897/zookeys.183.2834 PMC3332026 22573947

[B8] Sasa M Kikuchi M (1995) Chironomidae (Diptera) of Japan. University of Tokyo Press, Tokyo, 333 pp.

[B9] Sasa M Suzuki H Sakai T (1998) Studies on the chironomid species collected on the shore of Shimanto River in April, 1998. Part 2. Description of additional species belonging to Orthocladiinae, Diamesinae and Tanypodinae. Tropical Medicine 40: 99-147.

[B10] Sæther OA (1969) Some Nearctic Podonominae, Diamesinae and Orthocladiinae (Diptera: Chironomidae). Bulletin of the Fisheries Research Board of Canada 170: 1-154.

[B11] Sæther OA (1980) Glossary of chironomid morphology terminology (Diptera: Chironomidae). Entomologica scandinavica, Supplement 14: 1-51.

[B12] Sæther OA Sundal A (1999) *Cerobregma*, a new subgenus of *Polypedilum* Kieffer, with a tentative phylogeny of subgenera and species groups within *Polypedilum* (Diptera: Chironomidae). Journal of the Kansas Entomological Society 71: 315-382.

[B13] Sæther OA Oyewo EA (2008) Keys, phylogenies and biogeography of *Polypedilum* subgenus *Uresipedilum* Oyewo etSæther (Diptera: Chironomidae). Zootaxa 1806: 1–34. http://www.mapress.com/zootaxa/2008/f/z01806p034f.pdf

[B14] Sæther OA Andersen T Pinho L Mendes H (2010) The problems with *Polypedilum* Kieffer (Diptera: Chironomidae), with the description of *Probolum* subgen. n. Zootaxa 2497: 1–36. http://www.mapress.com/zootaxa/2010/f/zt02497p036.pdf

[B15] Townes HK (1945) The Nearctic species of Tendipedini. (Diptera: Tendipedidae (= Chironomidae)). American Midland Naturalist 34: 1-206. doi: 10.2307/2421112

[B16] Walker F (1856) Insecta Britannica, Diptera. Volume 3.Reeve & Benham, London, xxiv + 352 pp.

[B17] Zhang RL Wang XH (2004) *Polypedilum (Uresipedilum)* Oyewo and Sæther from China (Diptera: Chironomidae). Zootaxa 565: 1-38.

